# Lifelog Data-Based Prediction Model of Digital Health Care App Customer Churn: Retrospective Observational Study

**DOI:** 10.2196/22184

**Published:** 2021-01-06

**Authors:** Hongwook Kwon, Ho Heon Kim, Jaeil An, Jae-Ho Lee, Yu Rang Park

**Affiliations:** 1 Department of Biomedical Systems Informatics College of Medicine Yonsei University Seoul Republic of Korea; 2 Department of Information Medicine Asan Medical Center College of Medicine, University of Ulsan Seoul Republic of Korea; 3 Department of Emergency Medicine Asan Medical Center College of Medicine, University of Ulsan Seoul Republic of Korea

**Keywords:** churn prediction, digital health care, life-log data, topic modeling, recurrent neural network, deep learning interpretation, attribution method, integrated gradients, digital health, prediction, model, data, app, observational, time-series, neural network

## Abstract

**Background:**

Customer churn is the rate at which customers stop doing business with an entity. In the field of digital health care, user churn prediction is important not only in terms of company revenue but also for improving the health of users. Churn prediction has been previously studied, but most studies applied time-invariant model structures and used structured data. However, additional unstructured data have become available; therefore, it has become essential to process daily time-series log data for churn predictions.

**Objective:**

We aimed to apply a recurrent neural network structure to accept time-series patterns using lifelog data and text message data to predict the churn of digital health care users.

**Methods:**

This study was based on the use data of a digital health care app that provides interactive messages with human coaches regarding food, exercise, and weight logs. Among the users in Korea who enrolled between January 1, 2017 and January 1, 2019, we defined churn users according to the following criteria: users who received a refund before the paid program ended and users who received a refund 7 days after the trial period. We used long short-term memory with a masking layer to receive sequence data with different lengths. We also performed topic modeling to vectorize text messages. To interpret the contributions of each variable to model predictions, we used integrated gradients, which is an attribution method.

**Results:**

A total of 1868 eligible users were included in this study. The final performance of churn prediction was an F1 score of 0.89; that score decreased by 0.12 when the data of the final week were excluded (F1 score 0.77). Additionally, when text data were included, the mean predicted performance increased by approximately 0.085 at every time point. Steps per day had the largest contribution (0.1085). Among the topic variables, poor habits (eg, drinking alcohol, overeating, and late-night eating) showed the largest contribution (0.0875).

**Conclusions:**

The model with a recurrent neural network architecture that used log data and message data demonstrated high performance for churn classification. Additionally, the analysis of the contribution of the variables is expected to help identify signs of user churn in advance and improve the adherence in digital health care.

## Introduction

Customer churn prediction is one of the most important concerns for almost every company. If customers leave, then sales are reduced, and new customers are needed to replace the previous ones [1]. However, the cost of attracting new customers is 5- to 10-times higher than the cost of retaining customers [2]; therefore, it is much more effective to predict potential churn and prevent these customers from leaving by utilizing promotions or marketing.

Digital health care refers to public health services ranging from simple weight management to professional medicine using mobile devices [3]. With smartphone use becoming more common, the digital health care industry is growing, and numerous health-related apps have been launched [4]. This has provided many people with convenient access to digital health care; however, for them to achieve actual improvements in health, they need to use the apps consistently [5,6]. Therefore, the prediction of churn and the retention of digital health care service customers have significant implications for companies and for users.

Because of the importance of predicting customer churn, studies [7,8] have been performed. However, these studies have generally been conducted using statistical methods and time-insensitive machine learning techniques (eg, decision tree, logistic regression, or support vector machine) [7], by which some information can be lost during the preprocessing sequence [8]. Therefore, a model structure that can accept time-series patterns is necessary.

Many studies have used structured information about customers, which is generally stored in customer relationship management databases [9]. However, more customer text data are becoming available, such as online posts and messages, and it is known that analyzing text data improves predictive performance in customer churn problems [10,11].

We aimed to apply a recurrent neural network structure to leverage time-series patterns in user lifelog data and text message data to predict user churn for digital health care apps. We also aimed to examine the impact of time-series data on model performance and whether the presence of text data affects the performance of churn prediction.

## Methods

### Health Care App

This study was conducted using data from Noom (Noom Inc), a global digital health care app that provides lifestyle-related functions, such as food logs, exercise logs, weight logs, in-app group activities, and in-app articles. Users are encouraged to log their food intake, exercise every day, and record their weight every week [12]. Users of this digital health care service can also send messages to personal coaches to ask questions about dietary intake, exercise, mindset, or program descriptions. Personal coaches offer feedback to users, in the form of praise, emotional support, encouragement, and validation, based on the user's entries [12].

### Study Design

This study predicted customer churn based on lifelog data provided during the customer’s use period. Both paid and free services are available in this digital health care app, and the study defined customers who were refunded for their paid services as churn users.

We received anonymized and unidentified payment information and log data from the company for Korean users only. User data were randomly selected among users who had service payment records between January 1, 2017 and January 1, 2019. Users can select a program lasting 4, 8, 16, or 24 weeks; we limited our analysis to the 16-week program because the largest number of customers chose this period. Additionally, the service use period of the program varied depending on the date of payment and whether the users churned; therefore, we used only log data corresponding to each user’s paid service period for analysis.

Because the proportion of customers who request a refund is generally very small compared with the proportion of total users, the same ratio of retained users was extracted to match the number of churn users to address the problem of data imbalance associated with machine learning [13]. First, users who cancelled their subscriptions, with the refund date recorded, were selected. If their refund occurred after the end of the 16-week program, users were considered retained users and were excluded from the churn user group. The paid program includes a trial period lasting 7 days; during that time, users can request a refund after the initial payment. Therefore, users who received refunds during the trial period were excluded from the churn group because data for fewer than 7 days are insufficient for analysis reliability and generalization. Finally, 934 users were included as the churn group, and 934 retained customers were randomly selected for inclusion as the retained group; the 2 groups were equally matched with respect to gender [14].

This study was approved by the institutional review board (2017-1253) of the Asan Medical Center. The need for informed consent was waived because this study used routinely collected log data that were anonymously managed at all stages, including during data cleaning and statistical analyses.

### Model Structure

**Figure 1:**
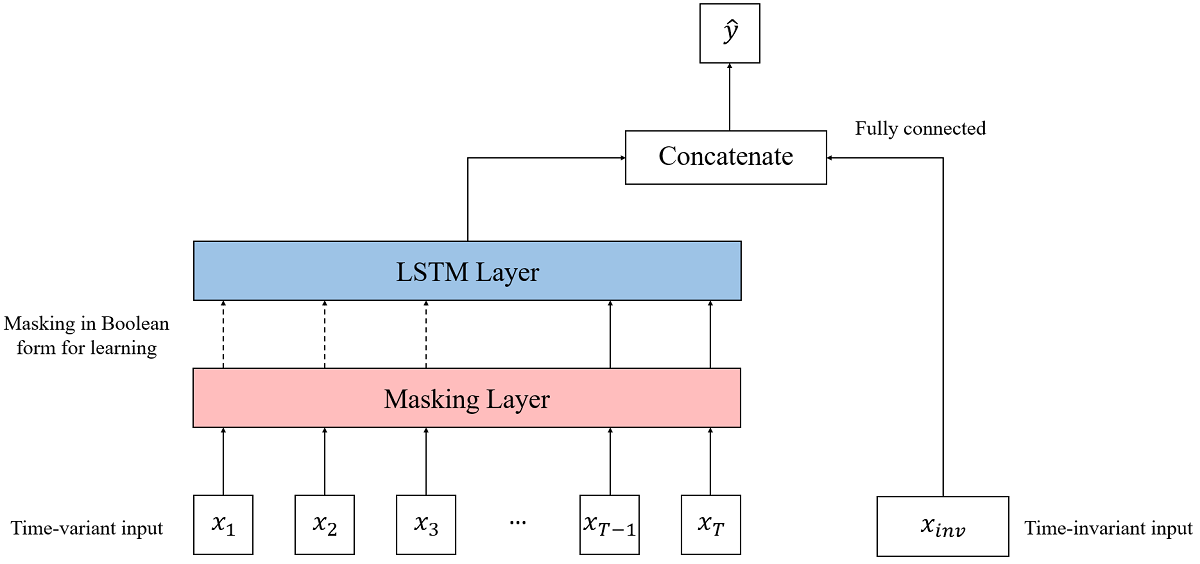
Overall model architecture. LSTM: long short-term memory.

The overall predictive model structure was designed to include both time-variant and time-invariant inputs. Inputs that occur over time include lifelog data such as step records, daily weight measurement records, diet intake records, and user text messages. Inputs that were considered constant included age, sex, initial BMI, or target BMI of each user (Figure 1).

The time-variant node used a long short-term memory structure, which belongs to the recurrent neural network family and is good for processing time-series data. Long short-term memory was developed to solve the vanishing gradient problem that can occur when training a basic recurrent neural network [15,16]. Long short-term memory has a memory cell that contains a node with a self-connected recurrent edge of a fixed weight, thereby ensuring that the gradient can exist over long time steps without vanishing or exploding [17].

Long short-term memory, as in other recurrent neural network-based models, should have the same sequence length input for time; therefore, the time steps of every sample need to be adjusted to ensure that they are the same. The period of service use was constant for retained users; however, for those in the churn group, the period of service varied depending on the time of departure of the user. Therefore, zero padding was used to lengthen the service period data of the churn group to 16 weeks.

One possible critical problem is that the day data of churn users are padded with zeros. Another is that the actual existing data of churn users are relatively short compared with the data of the retained users. If our algorithms notice that part of the sequence is padded with zeros (ie, in the case of churn users), then the problem of leaking the actual labels occurs. A masking layer was added to keep the model unaware of the length of the input sequence. The masking layer produces a Boolean-type tensor for learning whether to use certain values or to ignore them in downstream tasks, and it is used to handle time-series data of different lengths [18].

After padding and masking, time-variant inputs were processed in the long short-term memory layer and the outputs of the layer were concatenated with the time-invariant inputs. Finally, binary classification of the churn was performed.

### Text Message Preprocessing

The Noom app provides a function for users and coaches to communicate with each other through text messages. The messages that users receive from coaches can vary depending on their assigned coach; therefore, only messages sent by users were included in this study. User intentions can be more directly inferred from text messages than from log data. To input text message data in the model, the messages were vectorized. Although there are many advanced word-level and sentence-level embedding techniques, topic modeling with latent Dirichlet allocation was used in this study.

Topic modeling assumes each document is a set of random topics and probabilistically presents the importance of the topics and words in the document [19]. Latent Dirichlet allocation was used to estimate the probability that a word corresponds to a particular topic and the probability that a particular topic exists to find the topics in each unstructured document [20]. After topic modeling, each topic name was labeled as intended by the researcher (ie, the name of the topic is not determined by any criteria, but rather by discretion), taking into account the distribution of words corresponding to each topic. Topic modeling has the advantage of easy implementation and intuitive interpretation by examining the proportion of topics in sentences. For each text message, topic modeling was conducted to demonstrate the distribution of the topic proportion of each message. Additionally, since one person could send multiple messages during 1 day, the means of topic vectors was calculated to check the overall topic of the day, and the maximum of the vectors was taken to ensure that important topics that do not appear frequently were not diluted in the average.

Although topic modeling is popular because it is simple to use and easy to understand, there is a limitation: researchers must determine the optimal number of topics present in the documents [21]. This is not a serious problem if the exact number of topics is known, but it is very difficult to select the optimal number of topics without prior knowledge. To solve this problem, the optimal number of topics was determined using the coherence score, which calculates the similarity between the words included in the topic and calculates whether the topic consists of words that have semantic similarity [22].

### Interpretation Method

Identifying signs of churn in advance is extremely important for a company; however, because of its nested nonlinear structure, most deep learning models, including our model, are black boxes that, despite their good performance, do not provide information regarding the basis of the predictions [23]. To compensate for this uninterpretability, integrated gradients, which are part of an attribution method, were applied.

Attribution methods produce explanations of an analytic model by assigning an attribution value (relevance or contribution) to each input feature [24]. We used integrated gradients—a final prediction is calculated by multiplication of each variable and coefficient, and the output of the model is also a product of inputs and gradients in the deep learning structure, similar to linear regression [25]. The integrated gradients method was designed to improve the simple gradient approach, which does not satisfy implementation invariance (ie, the attributions are always identical for 2 functionally equivalent networks). Integrated gradients attempt to capture the effects of nonlinearities by computing the gradient along a line between the input data and given reference baseline data [26]. The integrated gradient of the dimension *i* is defined as follows:


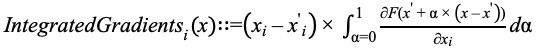


where


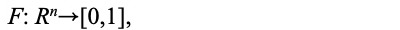


which represents a deep neural network function; x ∈ *R^n^* represents the input; and *x'* ∈ *R^n^* represents the baseline data (eg, zero-embedding vector for text neural network) [27]. Using this method, we can examine the effect of each variable on the final output of the model; to explain the effect of each variable on the model, we investigated the average value of the integrated gradients for each variable.

### Model Evaluation

During the model performance evaluation, we compared the performance from 2 perspectives: (1) how the performance of the model changes as data close to the point of churn (or retention) is determined at the last date, with some daily data excluded sequentially from the end of the period and (2) the performance differences in the model depending on the data regarding the presence of the text message vectors at every time point. Classification accuracy, F1 score, and area under the receiver operating characteristic curve were calculated.

This study was implemented with Python programming language (version 3.6.8). Data preprocessing procedures related to topic modeling were implemented using the gensim package (version 3.8.3). All neural network modeling including long short-term memory and masking layer were implemented using TensorFlow (version 1.14.0) and Keras (version 2.3.1).

## Results

### Baseline Characteristics

A total of 1868 eligible users (934 churn and 934 retained), were included in this study. The churn and retained groups showed no statistical differences in gender (*P*>.999) and age (*P*=.20). Both groups comprised mostly women (both groups 825/934, 88.3%), and the mean age was approximately 31 years. The initial BMI and target BMI were calculated based on the height, initial weight, and weight targeted by service use, and both showed no statistically significant differences (*P*=.41 and *P*=.19, respectively). Statistically significant differences were found for the total service period (*P*<.001) and the number of input days for meals (*P*<.001), messages (*P*<.001), walking (*P*<.001), and weight tables (*P*<.001), which are time-variant log data (Table 1).

**Table 1:** Demographic characteristics.

Variables		Churn users (n=934)	Retained users (n=934)	*P* value
**Gender, n (%)**				>.999
	Female	825 (88.3)	825 (88.3)	
	Male	109 (11.7)	109 (11.7)	
Age (years), mean (SD)		31.3 (7.1)	31.7 (7.9)	.20
Initial BMI, kg/m^2^, mean (SD)		25.2 (4.1)	25.1 (3.8)	.41
Target BMI, kg/m^2^, mean (SD)		22.0 (3.6)	21.8 (3.4)	.19
Total number of days of service use, mean (SD)		43.9 (31.3)	112.0 (N/A^a^)	<.001
Meal input days		24.0 (23.2)	53.8 (35.8)	<.001
Message sent days		11.8 (11.1)	22.3 (16.9)	<.001
Walk days		40.3 (29.5)	82.9 (32.9)	<.001
Weigh-in days		9.2 (11.4)	20.6 (19.9)	<.001

^a^N/A: not applicable.

### Topic Modeling

**Figure 2:**
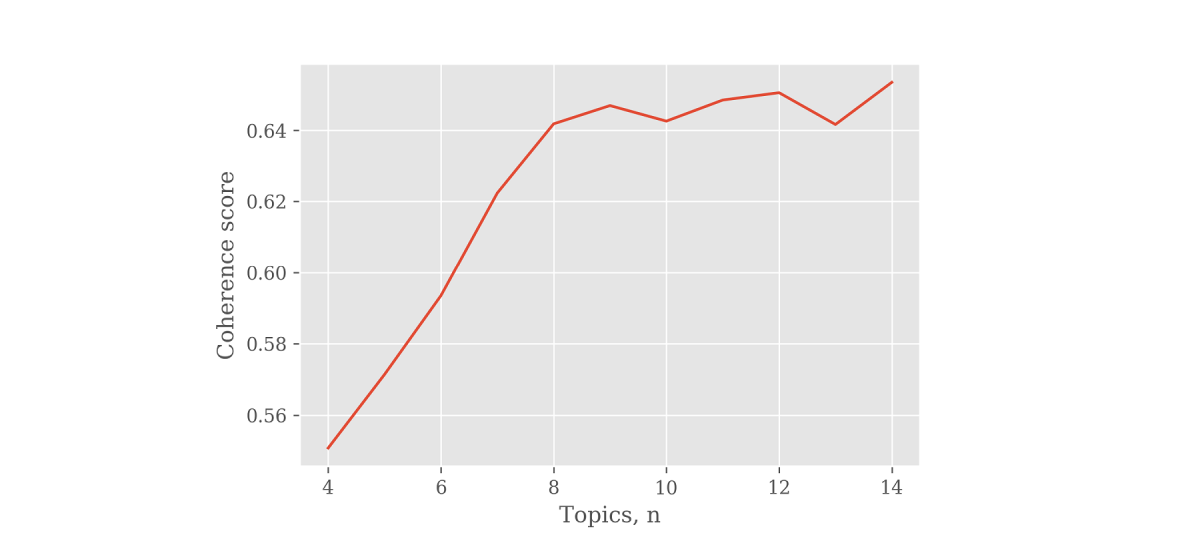
Coherence score by the number of topics.

The value of coherence according to the number of topics is shown in Figure 2. The scores steadily increased for up to 9 topics (0.6469); then, they repeatedly fluctuated and did not show much further increase. Therefore, the optimal number of topics was determined to be 9.

The results—the weighted proportion of the top 10 words—of topic modeling with 9 topics are shown in Table 2.

**Table 2:** Topic modeling results using a combination of the top 10 keywords and weight.

Top 10		Topic								
		Food plan	Schedule	Dining plan	Bad habit	Weight management	Daily life	Dietary intake	General exercise	Coaching
**Rank 1**										
	Keyword	Dinner	Today	Record	Water	Weekend	Time	Thought	Exercise	Thanks
	Weight	0.079	0.163	0.088	0.042	0.068	0.085	0.067	0.282	0.114
**Rank 2**										
	Keyword	Today	Yesterday	Tomorrow	Alcohol	Weight	Morning	Effort	Muscle	Diet
	Weight	0.032	0.065	0.05	0.036	0.065	0.074	0.052	0.021	0.032
**Rank 3**										
	Keyword	Meal	Next	Hello	Weirdness	Coach	Dinner	Control	Squat	Health
	Weight	0.031	0.027	0.028	0.035	0.064	0.059	0.051	0.02	0.026
**Rank 4**										
	Keyword	Snack	Saturday	Day	Weight	Goal	Lunch	Calorie	Stretching	Preparation
	Weight	0.03	0.025	0.028	0.026	0.052	0.05	0.049	0.02	0.026
**Rank 5**										
	Keyword	Promise	Start	Travel	Binge	Weight reduction	Meal	Food	Home	Coach
	Weight	0.03	0.024	0.023	0.025	0.037	0.04	0.044	0.017	0.023
**Rank 6**										
	Keyword	This time	Mission	Meal	Late-night meal	Height	Leave work	Diet	Aerobic	Stress
	Weight	0.028	0.023	0.02	0.017	0.036	0.02	0.043	0.013	0.022
**Rank 7**										
	Keyword	Home	Coach	Menu	Mind	Now	Company	Maximum	Gym	Body
	Weight	0.025	0.021	0.018	0.015	0.02	0.018	0.016	0.012	0.021
**Rank 8**										
	Keyword	Plan	Sunday	Eat out	Stamina	This time	Home	Intake	Feeling	Finish
	Weight	0.025	0.02	0.017	0.014	0.017	0.016	0.014	0.012	0.021
**Rank 9**										
	Keyword	Weekend	Action	Business dinner	Night	Hello	Possible	Fat	Week	Concern
	Weight	0.025	0.02	0.015	0.014	0.016	0.015	0.013	0.012	0.02
**Rank 10**										
	Keyword	Salad	This time	Person	Input	Management	Go to work	Meal	Start	Habit
	Weight	0.021	0.02	0.014	0.014	0.016	0.012	0.013	0.011	0.02

### Model Performance

The results shown in Table 3 show the classification accuracy, F1 score, and area under the receiver operating characteristic curve for the same test data when (1) some daily data were excluded sequentially from the end of the period and (2) with or without the text message vectors at every time point.

The results showed that the performance gradually increased as more data included the latter time. There was a performance difference of approximately 0.12 (F1 score, with text vector) when the whole-period data were included compared to when the last week data were excluded. The classification performance was generally better if text was included at almost all time points. When text data were included, the predicted performance increased by a mean of approximately 0.085 (F1 score), at every time point.

**Table 3:** Performance comparison.

Data period and text inclusion		Accuracy	F1 score	AUROC^a^
**Excluding the last week**				
	Without text vector	0.70	0.68	0.70
	With text vector	0.78	0.77	0.77
**Excluding the last 5 days**				
	Without text vector	0.70	0.68	0.71
	With text vector	0.79	0.78	0.78
**Excluding the last 3 days**				
	Without text vector	0.71	0.70	0.72
	With text vector	0.80	0.79	0.80
**Including full duration data**				
	Without text vector	0.83	0.83	0.82
	With text vector	0.89	0.89	0.89

^a^AUROC: area under the receiver operating characteristic curve.

### Model Interpretation

According to the results, the contribution of the *steps per day* variable, which denotes that the number of daily steps collected automatically, not those input by the user, was larger than the contributions of the other variables (0.1085). This was followed by *afternoon snack cal* (calorie intake from snacks during the afternoon) at 0.0999 and *receive sent ratio* (ratio of the received messages to the sent messages) at 0.0967.

**Figure 3:**
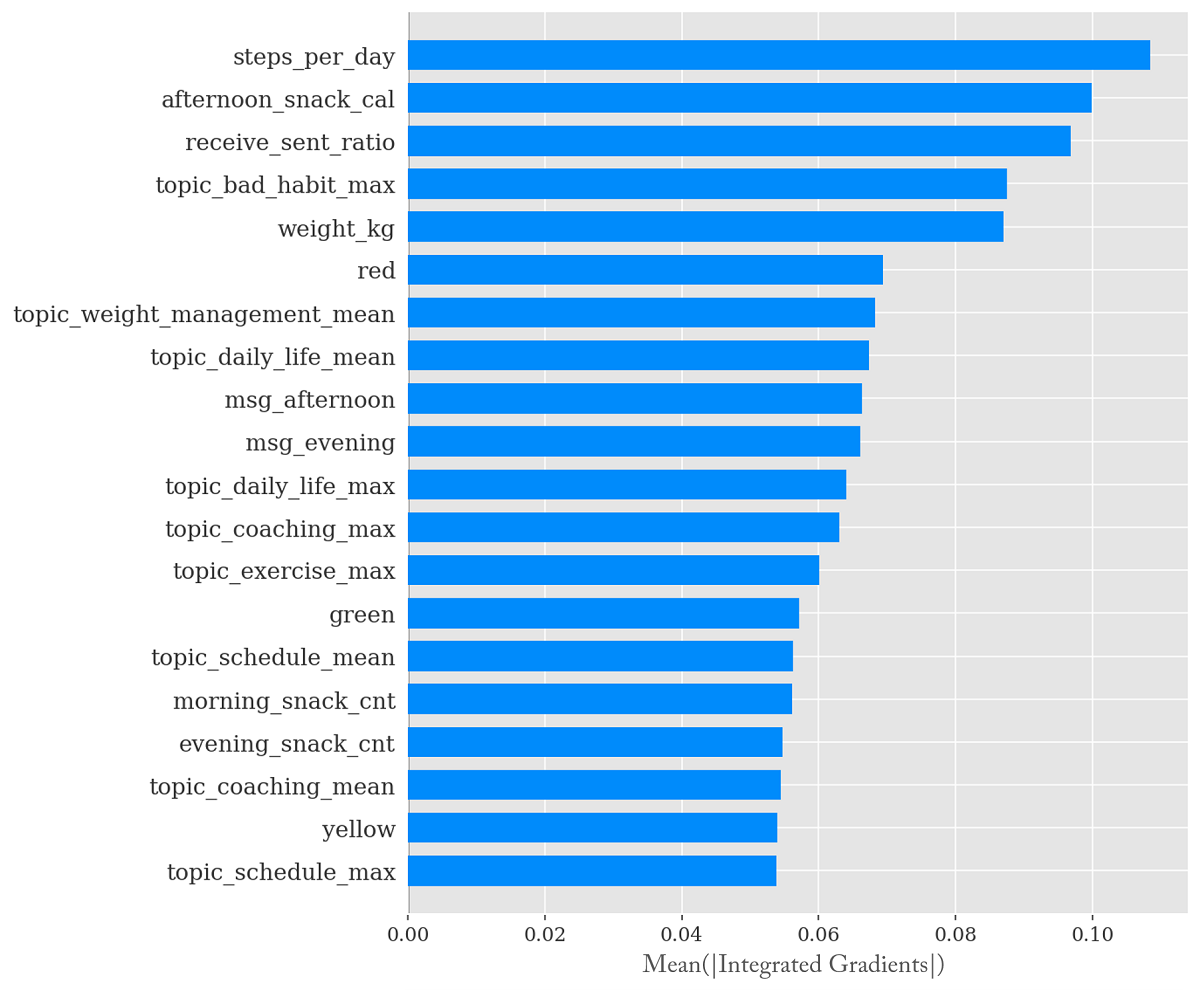
Average impact on model output of each variable.

Among the top 20 variables, there were 12 variables related to text messages, 6 variables related to meals, 1 variable related to walking, and 1 variable related to weight. Among the 9 topic vectors, *topic bad habit max*, which corresponds to poor lifestyle patterns (such as drinking alcohol, overeating, and late-night eating) showed the highest contribution (0.0875) (Figure 3).

**Figure 4:**
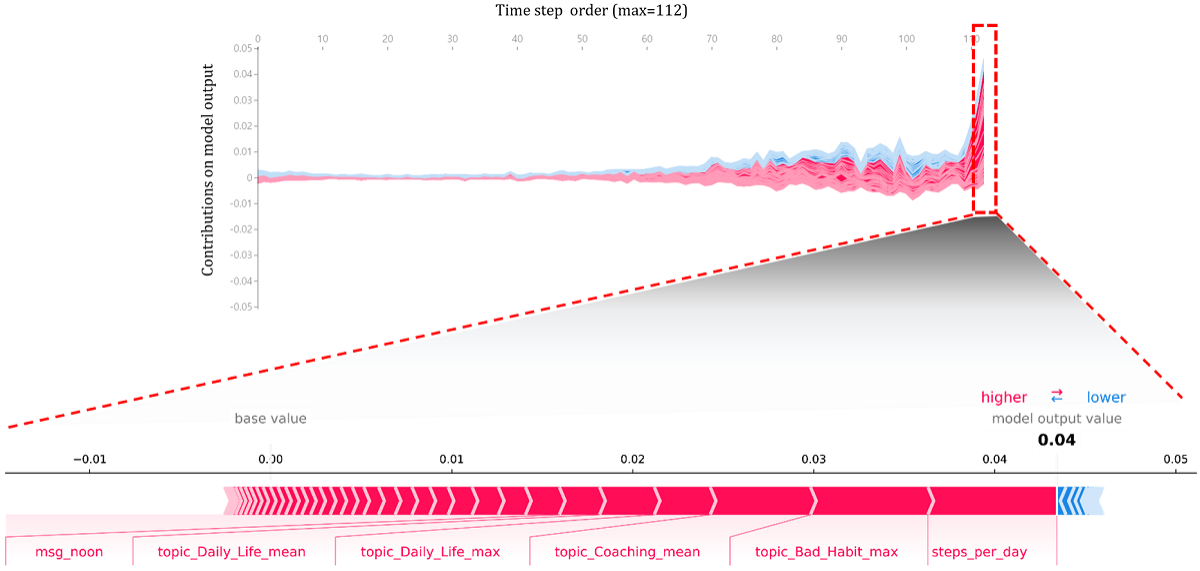
Churn users’ daily average contribution of variables over time (above, line plot) and contributions of each variable on the last day (below, bar plot).

**Figure 5:**
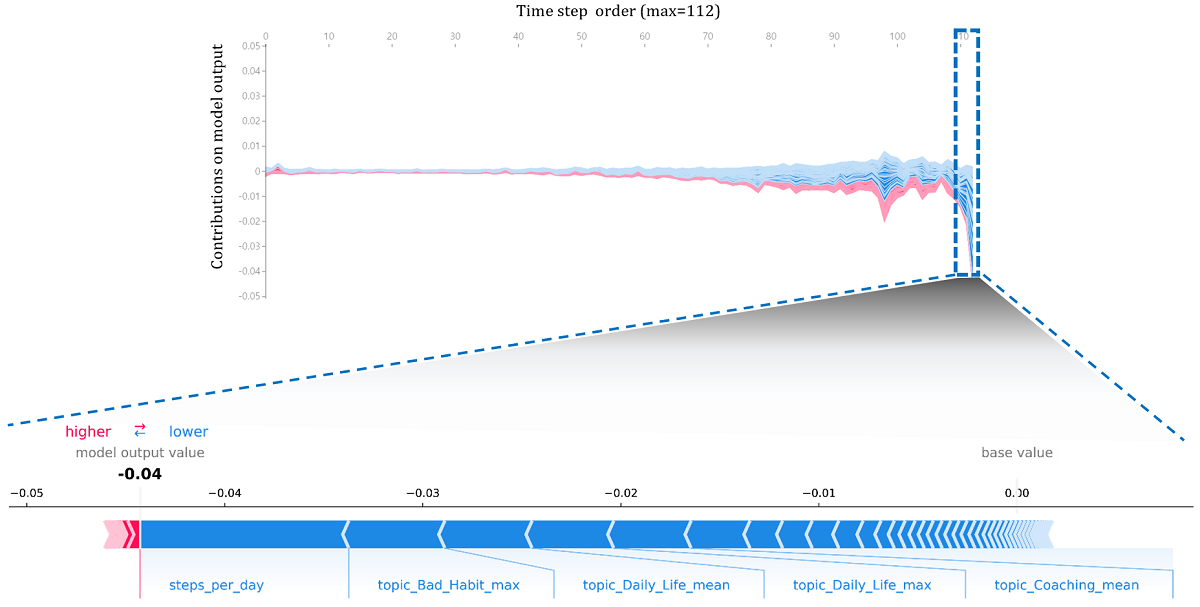
Retained users’ daily average contribution of variables over time (above, line plot) and contributions of each variable on the last day (below, bar plot).

To verify the contribution of each variable to the churn prediction model over time, the contribution of the variables corresponding to each time over 112 days (for the 16-week program) is expressed in line plots in Figures 4 and 5 (using force_plot [28]). Overall, the contributions of the variables appeared larger as they approached the later time points of churned and retained users.

**Figure 6:**
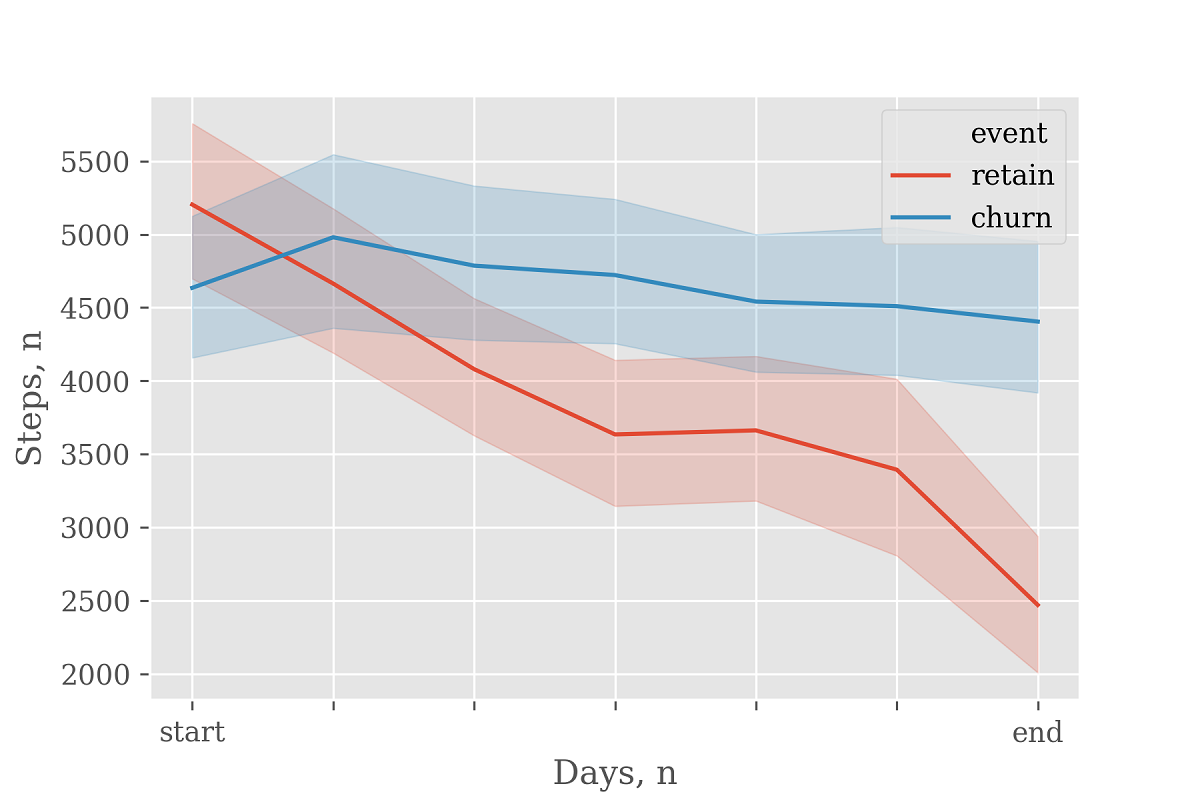
Changes in the mean number of steps_per_day from start to end.

Because the model in this study outputs the probability of churn, the graph increased to make the probability of churn high for churn users and low for retained users to decrease the probability of churn. Box plots show the contribution of the variable on each date. For example, when we check each graph of the last day’s churn and retained users showing the largest contributions, *steps per day* showed the greatest value, as seen for the overall variable contribution (Figure 6). Although several topic variables appeared for both churn and retained users, such as *bad habit*–related and *coaching*-related variables, the impact of each variable on the predicted performance was in the opposite direction.

To determine if the high contributing variables also showed differences in actual values between the 2 groups (churn and retained users), we checked the actual values of the *steps per day* variable, which had the greatest contribution in Figure 3. For retained users, the graph showed the entire 112 days; however, for churn users, the service use period varied depending on the user; therefore, the data were selected at varying intervals suitable for each user’s start and end dates. Comparisons of actual values showed that the churn group had maintained a relatively constant value or that the value decreased slightly from the initial date to the last date of service use (from 4774.02 to 4532.35). However, the value of the retained group had a tendency to continue to decrease by half (from 5048.37 to 2485.48) starting from a value at the time of initial service use that was similar to that of the churn group (Figure 6).

## Discussion

### Principal Findings

First, as evidenced by the model performance comparisons, for the end time-point, when there were more data, the predictive performance increased for churn and retained users. This could also be seen during the interpretation stage: the closer the end point, the higher the contribution of variables. A previous study [7] compressed the time-series data to input them in models that do not receive time-series patterns. Even in studies [29,30] using a model that could receive time-series data as input, data were lost when inputs were reduced (ie, data cut-off) in order to match the input shape of the models. In these cases, the performance of the model may be relatively poor because it does not reflect the fact that as the end of the period is approached, there is a greater difference between the churn and retained groups [31,32]. However, in our study, by including all the data for different durations of service use by each customer, our model was able to identify churn or retained patterns of the entire time period of service use, thereby improving the predictive performance. The ability to use data of different time lengths allows for the consideration of significant characteristics of the last moment and the immediate training and prediction of churn using only the data collected at that time without waiting for a certain period of time to collect data of equal lengths from all users. This makes it possible to promptly find users who are expected to churn. Therefore, companies can focus on such users and intervene, possibly improving user adherence to their mobile app. Because the steady use of mobile health care apps is closely related to improvements in the users’ health [33,34], increasing adherence is crucial to enhance satisfaction with the mobile app and for practical health promotion. Therefore, churn predictions with different lengths of data are important not only for companies but also for users.

Second, including text data can also provide better predictive performance. Because of the growing amount of unstructured customer data that can be collected both inside and outside a company, companies are studying unstructured data [35,36]. In particular, textual information can serve as important information for predicting churn [10]. Nevertheless, companies still struggle with extracting meaningful information from text [37]. In our study, we were able to increase the performance of churn prediction using vectors of messages sent by users as input to the model. We created message vectors through topic modeling to understand which specific topics affected the churn prediction by checking the contribution of each topic vector. For example, the topic that had the greatest impact on churn prediction was *bad habit topic* (eg, drinking alcohol, overeating, and late-night eating, which is known to have a negative influence on weight and health [38]). In other words, mentioning and conversing about these bad habits can also affect the churn of digital health care apps. With knowledge of this beforehand, companies can take measures to encourage the user not to churn by identifying and resolving the user’s problems or complaints. Therefore, adding text data not only increases the predictive performance of the churn predictive model but also enhances interpretability by providing intuitive understanding. Companies that want to identify signs of user churn in advance should consider collecting and analyzing unstructured data that directly project customers’ thoughts.

### Limitations

This study was conducted, using data from churn users until the day before the churn occurred and data for the entire service period of the retained users, as a proof of concept study. However, this assumption may not be appropriate in the real world because the company may not know when the customers will leave. Nevertheless, the probability distribution of the predictions for retained users tended to change the final prediction from churn to retention. In other words, the sensitivity of the churn user data are high; therefore, from the company’s point of view, the possibility of missing the churn user can be reduced. However, for practical forecasting by real-world companies, further studies of variations in probability of churn over time are needed. This study was retrospective. It used past records of mobile app users to identify signals of churn and identify the churn users. Because our analysis was retrospective, there was a constraint on its effectiveness for prospective data. Therefore, it is necessary to study whether early interventions for groups expected to be churn users provide more clinical indicators.

### Conclusion

We used a model with recurrent neural network architecture that used user log data and text data to determine the churn of digital health care users. Our analysis of variables is expected to help identify signs of user churn in advance and improve adherence in the field of digital health care.
